# Little bits of dragonfly history repeating exemplified by a new Pennsylvanian family

**DOI:** 10.1098/rsos.230904

**Published:** 2023-10-04

**Authors:** Nan Yang, Dong Ren, Olivier Béthoux

**Affiliations:** ^1^ College of Life Sciences, Capital Normal University, 105 Xisanhuanbeilu, Haidian District, Beijing 100048, People's Republic of China; ^2^ CR2P (Centre de Recherche en Paléontologie—Paris), MNHN, CNRS, Sorbonne Université, 57 rue Cuvier, CP48, 75005 Paris, France

**Keywords:** Odonata, evolution, venation, shape, Xiaheyan, new family

## Abstract

During its 320 Myr evolution, dragon- and damselfly (Odonata) wing morphology underwent intense modifications. The resulting diversity prompted comparative analyses focusing on phylogeny. However, homoplasy proved to plague wing-related characters. Concurrently, limited benefits were obtained from considering fossil taxa, similarly impacted. Herein, we investigate two aspects particularly affected by convergence, namely the acquisition of vein-like structuring elements derived from regular cross-venation, termed conamina; and the evolution of butter knife wing shape. Conamen implementation is found to be consistently linked with vein curvature sharpening, itself generating potential breaking points. Conamina therefore likely evolved to address wing integrity issues during ever-more-demanding flight performance. Moreover, an existing conamen is likely to trigger the acquisition of further, associated conamina. As for butter knife shape, previously documented in the extinct Archizygoptera and among damselflies, we report a new, 315 Ma occurrence with the rare species *Haidilaozhen cuiae* gen. et sp. nov. (family Haidilaozhenidae fam. nov.), from the Xiaheyan locality (China). The repeated acquisition of butter knife-shaped wing can be related to slow speed flight and, in turn, predator avoidance. In both cases of iterated regularities, the unique ‘network-and-membrane’ wing design proper to insects is found to compose a strong, constraining factor.

## 1. Introduction

Wing morphology occupies a central place in the systematics of damsel- and dragonflies (Zygoptera and Anisoptera; forming the Odonata), both extant and fossils. Indeed, this organ underwent intense transformations during the 320 Myr of the recorded evolution of the group [1–3], a phenomenon which can be related with flight performance, a key aspect in these insects' ecology, such as foraging and mating [4]. Actually, morphological transformations were so strong that it made it challenging to relate odonate wing venation with that of other insects until early representatives, displaying a yet generalized pattern, were discovered [5], putting an end to decades of debate [1,6–8].

This rich evolution is believed to have included numerous cases of homoplasy (see [9] among others). One prominent case regards the evolution of broad- versus narrow-winged forms [7 and references therein]. In the early twentieth century, the discovery, in Permian strata, of narrow-winged species lacking structures shared by all extant forms, such a closed discoidal cell, led early authors to assume that they were stem-Zygoptera and that the broad-winged Anisoptera had to have evolved from Zygoptera (so, itself regarded as a paraphyletic assemblage [10]). Later on, these ancient ‘zygopterous’ forms proved to represent a remote stem-group of Odonata, namely the Archizygoptera, to be excluded from the Panodonata [6,11,12]. This implied that narrow-winged forms appeared at least twice, in an extinct group during the Permian, and in Zygoptera, sometime during the Triassic [13]. More recent palaeontological evidence revealed the occurrence, as early as in the Pennsylvanian, of narrow-winged forms of yet unclear affinities [14–16], roaming among the more abundant and broad-winged griffenflies (including the iconic *Meganeura*).

Here, we describe a new case of narrow-winged form documented based on a very rare material composed of a sub-complete wing from the Pennsylvanian Xiaheyan locality, already known for its rich assemblage of griffenflies [17]. The well-constrained systematic placement of the new species attests to the early occurrence of a lineage of narrow-winged odonates distinct from the Archizygoptera. Investigating its relationships led to new considerations on the acquisition of structuring elements peculiar to odonates, and their evolutionary relevance.

## 2. Material and methods

### 2.1. Fossil material and its documentation

The newly described specimen is housed in the Key Laboratory of Insect Evolution and Environmental Changes, College of Life Sciences, Capital Normal University, Beijing, China (CNUB; Dong Ren, Curator).

Observations on the specimen CNU-NX1-466 were made using a stereomicroscope (Zeiss SteREO Discovery V8 stereomicroscope equipped with a pair of W-PL 10×/23 eye pieces, a Plan Apo S 1.0× FWD objective; all Zeiss, Jena, Germany). Photographs were taken using a Canon EOS 5DS digital camera coupled to a Canon MP-E 65 mm macro lens (both Canon, Tokyo, Japan) under polarized light (both polarizer and analyser). The resulting photographs were optimized using Adobe Photoshop CS6 (Adobe Systems, San Jose, CA, USA). The photograph reproduced in figure 2*d* was obtained by focus-stacking of four original photographs. Several photographs reproduced herein are composites, as indicated in figure caption. They could be a combination of photographs of both imprints of a specimen immersed in ethanol (‘eth-eth’ composites), or of one imprint under dry condition and then ethanol immersion (‘dry-eth’ composites). In the former case a dotted line (figure 2*a*) separates the areas known from both imprints versus from one imprint only. The final line drawing was prepared using Adobe Illustrator CS6 using both notes taken during observation and photographs. Faded sections indicate reconstructed elements.

**Figure 1.**
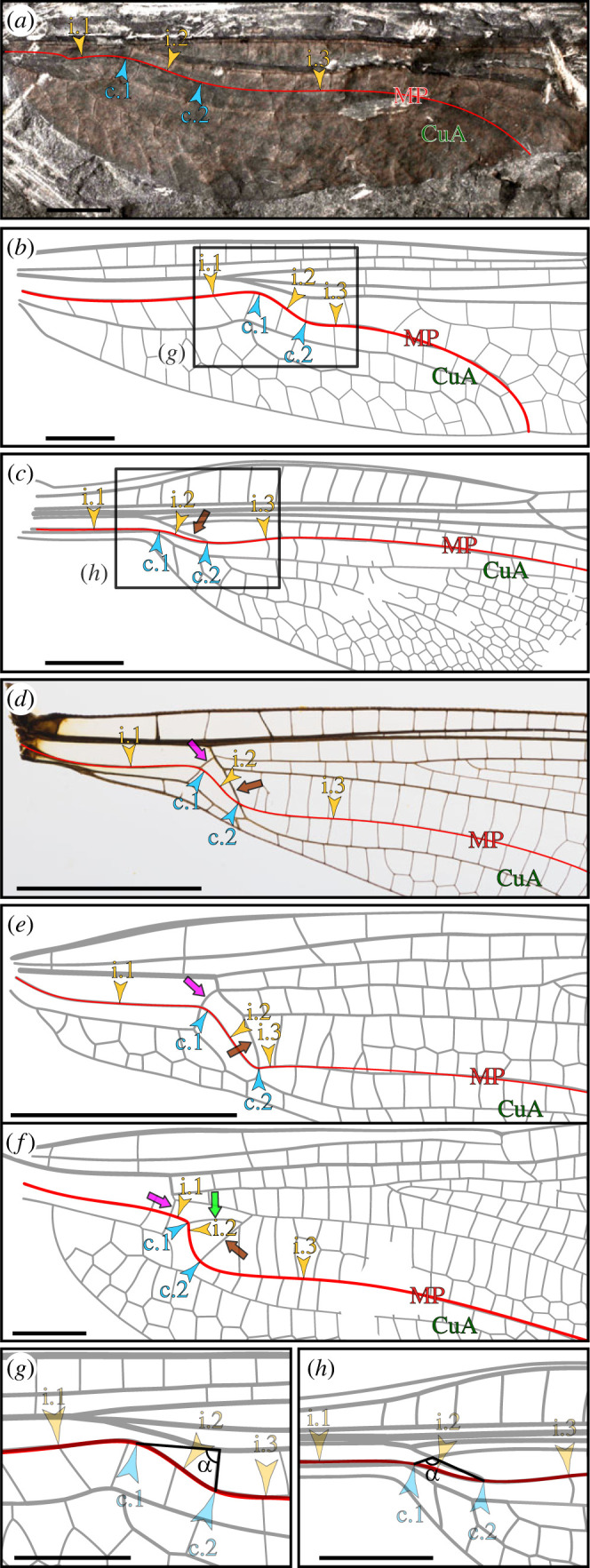
Critical points along the course of MP in Neodonatoptera and location of selected conamina (arrowhead, critical point; pons, brown arrow; parapons, magenta arrow; triangle conamen, green arrow). (*a*) Forewing of *Erasipteron larischi* Pruvost, 1933 [18] (holotype, specimen MB.I.455, Museum für Naturkunde Berlin; photograph courtesy A. Abele). (*b*) Forewing of *Polytaxineura stanleyi* Tillyard, 1935 [19] (redrawn from original description). (*c*) Forewing of *Triassologus biseriatus* Riek, 1976 [20] (modified from [21]). (*d*) Forewing of *Epiophlebia superstes* Selys, 1889 [22] (original photograph). (*e,f*) Fore- (*e*) and hindwing (*f*) of *Heterophlebia buckmani* (Brodie, 1845) [23] (redrawn from [24]). (*g,h*) Characterization of the first cross-vein in area between the posterior radial vein and anterior median vein (RP–MA area) either as a regular cross-vein (*g*; enlargement of *b*) or as the pons (*h*; enlargement of *c*), according to the angle made by (i) the segment connecting c.2 and the point of connection of this cross-vein on MA and (ii) the segment connecting c.1 and the point of connection of this cross-vein on MA (see text). Scale bars: (*a–e,g,h*) 5 mm, (*f*) 2 mm. i.1, i.2 and i.3, first, second and third inflexion points; c.1 and c.2, first and second points of maximum curvature; CuA, anterior cubital vein; MP, posterior median vein.

We carried out comparison with previously known species. Sources used to derive our comparative analysis (figure 1) are provided in electronic supplementary material, table S1. Early Odonata display a peculiar course of MP (in red on figure 1), previously characterized as ‘undulating’, or ‘forming a double curve’ (see [25,26] among others), and is most conspicuous in the Meganisoptera, including, among others, the iconic *Meganeura*. Besides its beginning and ending points, this course can be regarded as a Bézier curve with five critical points, in the following sequence from wing base to apex: a first inflexion point (i.1), a first point of maximum curvature (c.1), a second inflexion point (i.2), a second point of maximum curvature (c.2) and a third inflexion point (i.3). These points can be identified in all Neodonatoptera (figure 1) following a standardized sequence (electronic supplementary material, figure S2). A first approximation of the course of MP is a curve with the same starting and ending points as the vein itself (electronic supplementary material, figure S2*a*,*e*,*i*,*m*). The course of this curve shall minimize the area between the curve and the actual vein, and the discrepancy between the curve and the vein must be balanced. This curve crosses MP at several points but one in particular, once added and adjusted, allows an immediate improvement of the curve fitting onto the vein (electronic supplementary material, figure S2*b*,*f*,*j*,*n*): it is the second inflexion point (i.2), which might have originated earlier, in an evolutionary sense, than the others. In turn, two additional inflexion points can be readily identified: they are located at the points where the curve crosses MP immediately basal (for i.1), and distal (for i.3), to i.2. Once these points are adjusted, the curve fits tightly onto the vein (electronic supplementary material, figure S2*c*,*g*,*k*,*o*). At this stage, the points of maximum curvature (between i.1 and i.2, and between i.2 and i.3) can be confidently located (electronic supplementary material, figure S2*d*,*h*,*l*,*p*): if using a vector-based drawing software, they are the points where the sum of left and right handle lengths is minimal. In a more formal way, the curve sections between i.1 and i.2, and between i.2 and i.3, are cubic Bézier curves (as they have four control points—two points on the curve itself and the terminations of their respective handles). In turn, curvature maximum is formally located where the radius of the ‘osculating circle’ (a circle fitting inside the curve) is at its smallest.

**Figure 2.**
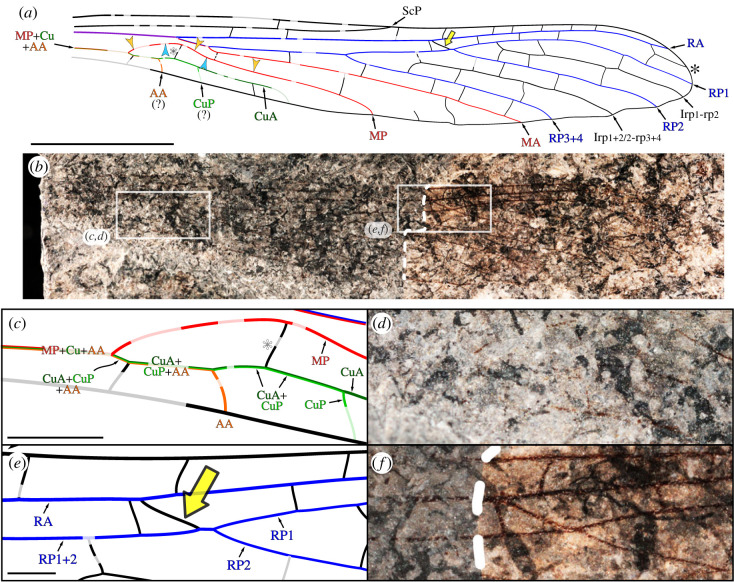
*Haidilaozhen cuiae* gen. et sp. nov., holotype specimen (CNU-NX1-466). (*a,b*) Overview. (*a*) Drawing of wing venation (black star, intercalary vein between RA and RP1; white star, first cross-vein in the area between MP and CuA(+CuP?); large yellow arrow, subnodus; arrowheads, figure 1). (*b*) Photograph (dashed line indicates the edge of the ‘b’ side, preserving apical half only; polarized light, basal half ‘eth-dry’ composite, distal half ‘eth-eth-dry’ composite). (*c,d*) Detail of wing base as located in (*b*). (*c*) Drawing. (*d*) Photograph (polarized light, under ethanol, focus stack). (*e,f*) Subnodal area, as located in (*b*). (*e*) Drawing. (*f*) Photograph. Scale bars: (*a,b*) 5 mm, (*c,d*) 1 mm, (*e,f*) 500 µm. AA, anterior anal vein; CuA, anterior cubital vein; CuP, posterior cubital vein; Irp_1_-rp_2_, intercalary vein between RP1 and RP2; Irp_1+2/2_-rp_3+4_, intercalary vein between RP1 + 2/2 and RP3 + 4; MA, anterior median vein; MP, posterior median vein; RA, anterior radial vein; RP1, anterior branch of RP1 + 2; RP1 + 2, anterior branch of posterior radial vein; RP2, posterior branch of RP1 + 2; RP3 + 4, posterior branch of posterior radial vein; ScP, posterior subcostal vein.

A reflectance transformation imaging (RTI) file was generated to document details of the right wings of the specimen MNHN.F.R51142 (Muséum National d'Histoire Naturelle, Paris, France; details of this technique are provided elsewhere [27]). It was derived from 54 photographs taken using a *ca* 50 cm diameter automated light dome driving the same camera body as specified above but coupled to a Canon 50 mm macro lens. Original photographs were optimized using Adobe Photoshop CS6 as before, itself achieved using the RTIbuilder software (Cultural Heritage Imaging). We provide an online Dryad dataset [28] containing this RTI file. Additional photographs of details were made using the setup as specified above.

### 2.2. Morphological terminology

Under the paradigm of the serial wing venation ground-plan [29,30], we use wing venation homology conjectures proposed by Riek & Kukalová-Peck [5] for Odonata. Corresponding abbreviations are: ScP, posterior Subcosta; R, Radius; RA, anterior Radius; RP, posterior Radius; RP1 + 2, anterior-most branch of the posterior Radius; RP1, anterior-most branch of RP1 + 2; RP2, posterior-most branch of RP1 + 2; RP3 + 4, posterior-most branch of the posterior Radius; MA, anterior Media; MP, posterior Media; Cu, Cubitus; CuA, anterior Cubitus; CuP, posterior Cubitus; AA, anterior Analis. Colour-coding is as follows: purple, R + MA (or RA + (RP + MA)); blue, radial system; red, median system; green, cubital system (dark green, CuA; light green, CuP; medium green, Cu); orange, anal system. To ease comparison between taxa (figure 4) we also colour-coded relevant areas, as follows: magenta, area posterior to CuA; turquoise, area posterior to AA. Particular points along the course of the MP vein also required abbreviations, as follows: i.1, i.2 and i.3, inflexion points 1, 2 and 3, respectively; c.1 and c.2, points of maximum curvature, 1 and 2, respectively. In figures 1 and 2, and electronic supplementary material, figure S1, inflexion points are indicated by yellow arrowheads; and points of maximum curvature by light blue arrowheads.

We propose to coin the general term ‘conamen’ (‘support’, ‘prop’, but also ‘exertion’ in Latin) to encompass the variety of specialized, strengthened cross-veins acquired in the course of Odonata wing morphology evolution. The most prominent of these conamina is the ‘pons’. The latter term was proposed by Deregnaucourt *et al.* [31] to replace the more widespread, but confusing term ‘MAb’ [1,3] (also called ‘distal discoidal cross-vein’ [1], or ‘discoidal cross-vein’ [32]). It is indicated by a large brown arrow in figure 1*c–f*. Fundamentally, it is a specialized cross-vein connecting the free portions of MA and MP (or MP at the point where it diverges from MP + CuA). To better characterize this element, we carried out a morphological comparative analysis considering the main groups of Odonata.

To further ease discussion, the term ‘parapons’ is proposed for the cross-vein preceding the pons in the MA–MP area. It closes basally the discoidal cell (also termed ‘quadrangle’ in Zygoptera [33]; in Anisoptera, in which the discoidal cell is split, it closes the ‘supratriangle’ [34], or ‘hypertriangle’ [3]). The parapons has also been termed ‘basal discoidal cross-vein’ [1], or considered to compose the posterior part of the arculus [32,35], and was first acquired in the hindwing of early relatives of both Zygoptera and Anisopera [1,3]. It is indicated by a large magenta arrow in figure 1*d–f*. In turn, we understand ‘arculus’ as the structure encompassing both RP + MA and the parapons, and, where applicable, a very short section of MA (between its divergence from RP + MA and its connection with the parapons itself).

Finally, the cross-vein splitting the discoidal cell into the hypertriangle (or ‘supratriangle’) and the discoidal triangle (or ‘triangle’) is called the ‘triangle conamen’, and is indicated by a large green arrow in figure 1*f*.

### 2.3. Phylogenetic and systematic framework

The phylogenetic and systematic framework adopted in this account is a compilation of consensual, largely congruent and recent accounts (see [1–3,11,25] among others).

## 3. Results

### 3.1. Comparative analysis

Our comparative analysis, carried out to better characterize the pons, led us to unravel a recurring pattern of acquisition of strengthened cross-veins (herein, ‘conamina’) in Odonata wing venation evolution. The first outcome of our endeavour is that the point where MP is at its maximum of ‘concave-up’ curvature (i.e. at c.2) in early stem-Odonata (figure 1*a*,*b*) can actually be found in crown-Odonata: it is located where MP and CuA diverge (figure 1*d–f*). In other words, the ‘double curve’ conspicuous in the extinct Meganisoptera is present in all Neodonatoptera. Another outcome is that in taxa showing a clear pons (brown arrow in figure 1), such as the extinct Triadophlebiomorpha (figure 1*c*) and in crown-Odonata (figure 1*d–f*), this structure is consistently connected to MP opposite its second point of maximum curvature (c.2).

We noted several cases of co-occurrence of a conamen and a point of maximum curvature. In a subset within Triadophlebiomorpha, two strengthened, aligned cross-veins, forming a structure termed the ‘pillar’, are located (i) in the CuA–CuP area and (ii) in the area between CuP and the posterior wing margin, respectively [31]. This structure can be extended by an additional strengthened cross-vein located between MP and CuA and which, incidentally, connects the pillar and the pons [36,37]. It is remarkable that these cross-veins connect to both CuA and CuP at points of maximum curvature along these veins. It is also relevant that no pillar occurs in Triadophlebiomorpha in which CuA and CuP have a more rectilinear course, as, for example, in *Triassologus biseriatus* (figure 1*c*) [21]. The pillar is therefore a conamen.

Nel & Pinet [3] defined the Odonata as the Odonatoptera possessing a closed discoidal cell in the hind wing. This closure is achieved by another conamen that we propose to term ‘parapons’ (magenta arrow in figure 1*d–f*; also called ‘basal discoidal cross-vein’ [1], ‘inner side of quadrangle’ [33]). It bridges, on the one hand, RP + MA at the point where its two constituent veins diverge and, on the other, MP at its point of maximum curvature c.1. It is notable that the parapons is often complemented by another conamen, aligned with it and located posteriorly to it, in the MP–Cu area (figure 1*d–f*).

A further demonstrative case is the cross-vein splitting the discoidal cell into the hypertriangle (or ‘supratriangle’) and the discoidal triangle (or ‘triangle’), characteristic of Anisoptera. When distinctly stronger than a regular cross-vein, this ‘triangle conamen’ (green arrow in figure 1*f*) bridges MA, at its point of maximum curvature, and MP. The development of the triangle conamen can be related to a forward shift of the c.1 point, resulting in a mismatch with the parapons. With the concurrent backward shift of c.2, the acquisition of the triangle conamen can be related, also, to a strong curvature sharpening.

Finally, these observations provide some clues as to characterize the pons. However, connection with c.2 is not self-sufficient. A key case is whether the cross-vein facing c.2 in Protanisoptera, such as *Polytaxineura stanleyi* (figure 1*b*), shall be regarded as a genuine pons, as some authors tentatively did [1,19] (but see [3,38]). Unlike in Triadophlebiomorpha and crown-Odonata (figure 1*c–f*), in which the pons is oblique, in alignment with RP + MA and MA, this cross-vein has a more regular habitus. Obliquity can be assessed by measuring the angle made by (i) the segment connecting c.2 and the point of connection of this cross-vein on MA and (ii) the segment connecting c.1 and the point of connection of this cross-vein on MA (figure 1*g*,*h*). For early forms, whether it equates to or is less than 90° (figure 1*g*) or greater than 90° (figure 1*h*) can provide a clear-cut characterization of the pons being absent (first case) or present (latter case). This criterion does not apply in more derived taxa, such as Anisoptera, in which c.2 acquired a very basal position (figure 1*f*). As defined, the occurrence of the pons in a number of taxa, including the Pennsylvanian, narrow-winged *Bechlya ericrobinsoni*, might have to be reconsidered.

### 3.2. Systematics

Order Odonata Fabricius, 1793

Family Haidilaozhenidae fam. nov.

**Diagnosis.** By monotypy, that of the type genus.

**Type genus.** *Haidilaozhen* gen. nov.

Genus *Haidilaozhen* gen. nov.

**Diagnosis.** By monotypy, that of the type species.

**Type species.** *Haidilaozhen cuiae* sp. nov.

**Etymology.** The genus name derives from the Chinese idiom literally translated as ‘finding a needle from the sea bottom’ (i.e. ‘finding a needle in a haystack’), referring to both the wing shape of the type-species and its rarity (hence the difficulty to find it; insect species are commonly sampled by tens of specimens, and for some by hundreds, at the Xiaheyan locality [39,40]); moreover, the sediments embedding the wing were deposited under a marine environment [41]; feminine in gender.

*Haidilaozhen cuiae* sp. nov.

(figure 2*a*–*f*; electronic supplementary material, S1)

**Diagnosis.** Wing narrow and long; subnodus present; intercalary vein present between RA and RP1; area between MA and MP large distally; occurrence of common MP + Cu + AA stem (i.e. AA fused with MP + Cu from the wing base); MP diverging from MP + Cu + AA obliquely; area between MP and CuA large, with a long, oblique cross-vein; free portions of AA and CuP very short.

**Material.** Holotype specimen CNU-NX1-466, two sides (polarity unknown).

**Etymology.** The species epithet honours Yingying Cui, for her contribution to the knowledge on fossil insects, including those from Xiaheyan.

**Description.** Specimen CNU-NX1-466 (holotype; figure 2*a*–*f*); two complementary imprints (polarity unclear, because of rock compression) of a nearly complete single wing (basal three fifths missing in one of the imprints); wing length 21.8 mm, width 3.5 mm; base narrow; several cross-veins between anterior wing margin and RA; ScP long and straight, reaching anterior margin near wing mid-length; R + MA thick; RA simple, with a slightly concave course distal to the end of ScP, and slightly convex in its distal two fifths, reaching anterior wing margin near the apex; occurrence of a particularly strong and oblique cross-vein (subnodus) between RA and RP, slightly before the RP1/RP2 fork (figure 2*e*,*f*); occurrence of a short intercalary vein between RA and RP1 close to apex (black star in figure 2*a*); RP + MA diverging from R + MA in the basal fifth of wing length, divided into RP and MA after a short distance; RP1 + 2/RP3 + 4 split located before wing mid-length; simple Irp_1_-rp_2_ (IR1) and Irp_1+2/2_-rp_3+4_ (IR2), almost straight; MA simple, obliquely reaching posterior margin at the distal two fifths; MA and MP connected by a short cross-vein close to MA origin, not particularly strong; MP, Cu and AA forming a single, basal common stem; MP diverging anteriorly from it and directed towards MA; MP simple, reaching posterior wing margin slightly distal to the RP1 + 2/RP3 + 4 split; first cross-vein in the MP–CuA area long (white star in figure 2*a*,*c*); AA diverging from CuA + CuP + AA basal to this cross-vein, very short; CuA + CuP 1.4 mm long before the (presumed) CuA/CuP split; CuP very short; CuA parallel to the posterior wing margin for most of its preserved length.

**Locality and horizon.** The holotype specimen was collected by Yuanyuan Peng at Peacock 1 excavation site in 2014, Xiaheyan locality (Ningxia Autonomous Region, Popular Republic of China); Yanghugou Formation; early Moscovian (Bolsovian), Middle Pennsylvanian [41].

**Figure 3.**
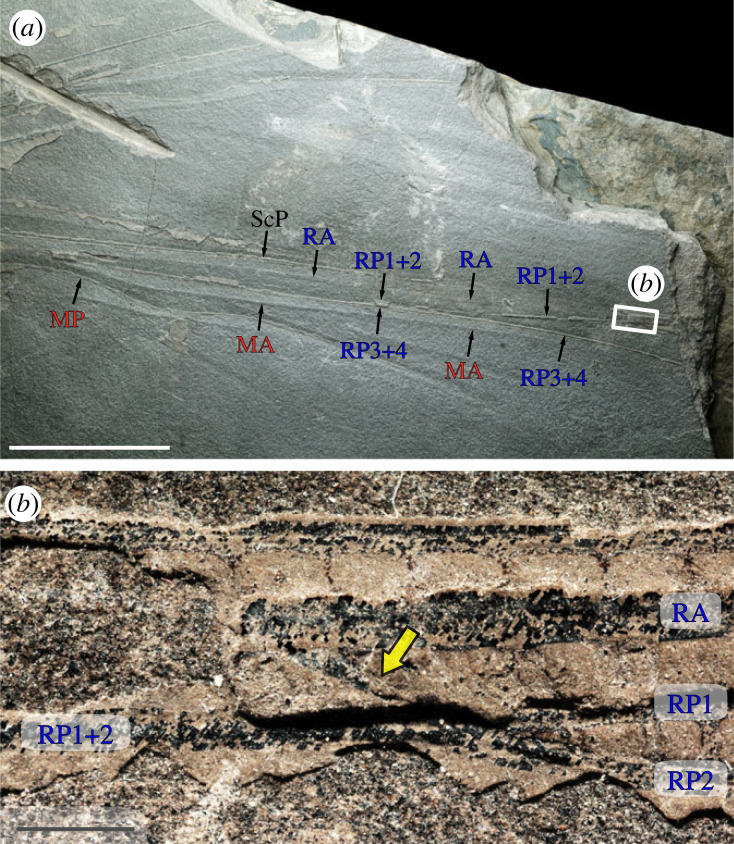
*Meganeura monyi* (Brongniart, 1884) [42], holotype specimen (MNHN.F.R51142). (*a*) Distal part of right wings (RTI extract). (*b*) Area of the subnodus as located in (*a*), photograph (polarized light, eth-dry composite; yellow arrow, subnodus). Scale bars: (*a*) 50 mm, (*b*) 2 mm. MA, anterior median vein; MP, posterior median vein; RA, anterior radial vein; RP1, anterior branch of RP1 + 2; RP1 + 2, anterior branch of posterior radial vein; RP2, posterior branch of RP1 + 2; RP3 + 4, posterior branch of posterior radial vein; ScP, posterior subcostal vein.

**Figure 4.**
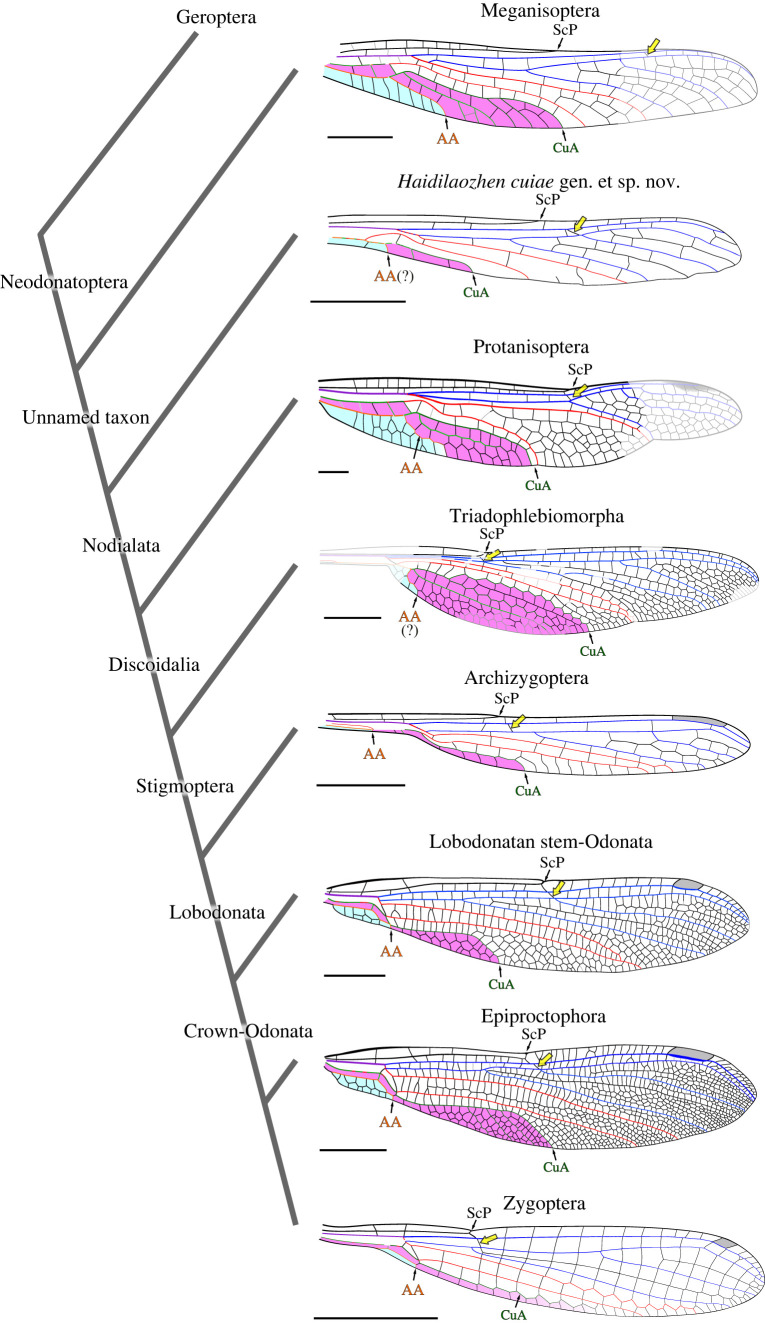
Wing shape in Odonata and stem-relatives in a phylogenetic context (yellow arrow, subnodus; magenta area, area between CuA, AA and the posterior wing margin; light blue area, area between AA and posterior wing margin). Butter knife wing shape appeared at least three times in the evolution of the group, in *Haidilaozhen cuiae* gen. et sp. nov., Archizygoptera and Zygoptera. Scale bars: 5 mm. AA, anterior anal vein; CuA, anterior cubital vein; ScP, posterior subcostal vein.

**Remarks.** The occurrence of a subnodus decisively indicates that the species to which the new specimen belongs is a stem-Odonata, as this structure is present in both Meganisoptera [17,26] (figure 3) and Nodialata (figure 4). This proposal is further corroborated by the branching pattern of RP, following a (2,1) pattern; the occurrence of intercalary veins between RP branches; and the occurrence of a RP + MA stem.

Several possible interpretations of the preserved venation pattern near the wing base can be considered (figure 1*c*,*d*; electronic supplementary material, figure S1). We favour a proposal implying that AA is basally fused with MP + Cu (at least in the preserved section), and that it diverges from this composite stem basal to the RA/RP + MA split (figure 1*c*; electronic supplementary material, figure S1*a*). One alternative interpretation predicts that AA diverges more basally (electronic supplementary material, figure S1*b*), but the orientation of the structure then interpreted as AA tends to contradict this proposal. A more deeply different alternative implies that AA is fused with the posterior wing margin near the wing base, diverges from it near the first fork of (then) MP + Cu, briefly fuses with CuA + CuP (or CuP), and then reaches the posterior wing margin again (electronic supplementary material, figure S1*c*). This proposal considers the fact that AA is distinct from MP + Cu in Meganisoptera and Protanisoptera, regarded as the closest relatives of the new species (figure 3). However, a re-emergence of AA (from the posterior wing margin) would be a transformation more radical than a mere fusion of AA with MP + Cu which, incidentally, is expected to have occurred in Triadophlebiomorpha and Archizygoptera (figure 4).

The course of CuA is also to be discussed. It could either run fused (i) with CuP and AA (figure 1*c*; electronic supplementary material, figure S1*d*), or (ii) with MP, until it would diverge from it and fuse with CuP (electronic supplementary material, figure S1*e*). Although a distal fusion of CuA and CuP is consistent with the configuration admitted for both Meganisoptera and Protanisopera (figure 4), it would necessitate CuA to be directed backwards at some point of its course, which is unlikely. Moreover, under the proposal (i), an oblique cross-vein occurs between MP and CuA (more specifically, CuA + CuP; white star in figure 1*c*, electronic supplementary material, figure S1*d*) at the point where MP is the closest to RP + MA. Such cross-vein is present in Protanisoptera (white star in electronic supplementary material, figure S1*f*), making this interpretation more parsimonious. It implies that CuA and CuP diverge fused together from MP + Cu(+AA) (as opposed to successively, as in most Meganisoptera and in Protanisoptera), but a similar configuration is known to have occurred in some Meganisoptera [8]. Overall, these aspects of wing venation homologies have very limited implications on the extent of the CuA and AA areas.

The new material differs from all currently known species of Odonata in that it has the following combination of morphological features: wing narrow; ScP terminates on anterior margin approximately at wing mid-length (a trait shared with some Meganisoptera); oblique strong cross-vein subnodus opposite the RP1/2 fork, slightly beyond mid-wing (in a more distal position in Meganisoptera); CuA reaching posterior margin near the basal third of wing length, the area between CuA and posterior margin being very narrow (CuA long and broad in Meganiscoptera and Protanisoptera); occurrence of a short intercalary vein between RA and RP (rarely present in Palaeozoic Odonata).

Further comparison with other, known Pennsylvanian narrow-winged morphotypes is necessary. Comparison with *Bechala sommeri* Ilger & Brauckmann [14] is a relevant step. Notably, the new material and this species share an intercalary vein between RA and RP1. However, the subnodus is located significantly closer to the wing apex in *Bechala sommeri*. Moreover, based on the preserved parts, it can be inferred that both CuA and CuP were much longer than in the new material. *Bechlya ericrobinsoni* Jarzembowski & Nel, 2002 [15] is another enigmatic, narrow-winged Pennsylvanian Odonata. In contrast with the new material, both ScP and the subnodus are located very basally (approximately in the basal quarter of the wing length) in this species. Notably, the subnodus is then located nearer to the RP1 + 2/RP3 + 4 split than to the RP1/RP2 split (as in the new material). Finally, CuA is more developed in *Bechlya ericrobinsoni* than in the new material. Finally, in *Sowiakala perprocera* Zessin, Brauckman & Leipner, 2021 [16], ScP ends more basally than in the new material, and the subnodus is seemingly absent. The vein CuA is also very developed. Further comparison is impeded by the available data. Indeed, as currently documented, the median and cubital systems in *Sowiakala perprocera* sharply contrast with the accepted Odonata wing venation ground-plan: notably, the origin of MP is challenging (it either diverges from R + MA, or is connected to R + MA by a strong cross-vein). Finally, all these narrow-winged species are larger than the new material (wing width of 3.5 mm in the new material; 5.2 mm in *Bechala sommeri*; 4.5 mm in *Bechlya ericrobinsoni*; about 5 mm in *Sowiakala perprocera*).

In summary, the new species cannot be placed into any possible known main groups of Odonata. It is then well justified to erect a new genus and a new family to accommodate the new species the new material belongs to.

## 4. Discussion

The multiple examples of repeated acquisitions of similarities independently from ancestry [43], i.e. convergences, suggests that, to an extent remaining to be evaluated, organismic evolution is constrained and therefore predictable. Documenting these instances is therefore fundamental to our understanding of evolution. That of wing venation in Odonata has been traditionally regarded as plagued by homoplasy [1,9,12], including convergences, and often related with mechanical constraints during flapping flight. It is not unexpected, given the foraging niche of aerial predators these insects occupy as well as their competitive mating process [4], requiring superior flight performances. Other aspects of Odonata locomotor behaviour, such as migratory flight, have also been advocated as drivers of convergence in wing venation [44].

### 4.1. Structuring elements of wing venation

A signature feature of Odonata wing venation evolution is the implementation of vein-like structuring elements derived from regular cross-veins, thickened and orientated obliquely, herein termed conamina. On the basis of our comparative analysis, we argue that the acquisition of these structures is the mere consequence of the migration of points of maximum curvature, resulting in curvature sharpening which, in turn, generate potential breaking points during wingbeat cycle. Conamina are therefore likely to act as props, structures driving tensile forces, or structures constraining wing deformability during stroke. For example, the pons, diagnostic of the Discoidalia, is clearly part of a strengthening structure, at least at its origin. It bridges the anterior median vein (MA) and the posterior median vein (MP) at a point of maximum curvature of the latter (c.2). Additionally, it is commonly more or less aligned with (i) the stem composed of the posterior radial vein and the anterior median vein (RP + MA) and (ii) a short section of MA (between its divergence from RP + MA and its connection with the pons itself; brown arrow in figure 1*c–e*). Therefore, a possible function of this organization is, during the downstroke phase of wingbeat, to drive tensile force exerted onto MP by the area it delimits towards a robust support, here the anterior radial vein (RA). It is relevant that RA itself is propped by antenodals (when present), themselves leaning against the anterior wing margin. Numerical simulations suggested that the pons, together with other elements of the ‘basal complex’, prevents the transfer of stress experienced during wingbeat towards the wing base [45], a function which is not excluding that of tensile force driver.

The parapons (magenta arrow in figure 1*d–f*) and the triangle conamen (green arrow in figure 1*f*), also involved in the ‘basal complex’, likely evolved to take a similar role at acting as props and/or driving forces generated during downstroke. Proximity with the pons suggests that these conamina compose implementations of this element. They may have evolved to address functional demands of secondary importance in the absence of a pons, but gaining a higher degree of relevance once this structure was acquired. In its earliest condition, the parapons is (i) located opposite a point of maximum curvature (i.e. a potential breaking point; c.1) and (ii) aligned with another, rectilinear structure, which can either be the free section of the posterior cubital vein (CuP; figure 1*d*,*e*) or another conamen (commonly, in hind-wing). The distribution of vein joint combinations in extant Odonata provides additional evidence of the supporting role of conamina. The pons is commonly connected with MP (i.e. at c.2) via a dorsal ‘bridge joint’ type, and the parapons via a dorsal ‘double-rigidly fused’ type, both of which being comparatively rigid; and, as for the ‘bridge joint’ type, with a very restricted distribution [46].

The parapons being aligned with the free section of CuP is one among several examples where a conamen is complemented by an additional prop aligned with it. In the extinct Triadophlebiomorpha, the ‘pillar’ is composed of two aligned conamina located in the area between the anterior and posterior cubital veins (CuA–CuP area), and the area between CuP and the posterior wing margin, respectively [31]; and it can be complemented by a further conamen located in the MP–CuA area [36,37], aligned with the pons. The resulting structure is roughly perpendicular to the posterior wing margin. In all crown-Odonata, CuA diverges from MP + CuA opposite the pons; so, in that sense, at the exact same point as the MP–CuA conamen of the Triadophlebiomorpha. More strikingly, in Polythoridae (banner-winged damselflies) and Calopterygidae (broad-winged damselflies), the pons and CuA form a rectilinear structure, also often perpendicular to the posterior wing margin [33]. Furthermore, in the latter group, CuA is either strongly bent posteriorly, or complemented by a reinforcing structure, which may be either a conamen or a posterior branch of CuA, as in *Calopteryx* spp. Similarity with the triadophlebiomorphan pillar is then realized.

In summary, reinforcements sharing very similar features evolved repeatedly in the wing postero-basal area, often in prolongation of the pons. And once acquired, a conamen is likely to be complemented by another structure. As far as systematics is concerned, caution is therefore to be exerted when resorting to conamina for classificatory purposes.

The ‘design problem’ of maintaining wing integrity during ever-more-demanding, high-performance flight was addressed by a limited number of solutions. Once the pons was acquired, it was complemented by a variety of structures, often orientated obliquely or transversally, in different lineages independently. An attempt can be made to characterize this function-related iterated regularity to assess whether it constitutes a ‘true convergence’ [47,48]. A key point is to conceive insect wings as essentially composed of a corrugated tubular network and a membrane connecting its elements, altogether enclosed by a tubular margin.

We would argue that, as for specificity, it is very precise, as it is focused on a particular wing area. Clearly, all observed reinforcing structures derived from cross-venation, or involved sections of main veins. Therefore, their iterated acquisitions most likely depend on a shared developmental mechanism. In that sense, these events are highly dependent. As for the scope of the case at hand, it is relevant to consider that locomoting in an atmosphere has been repeatedly addressed, on Earth, by the acquisition of movable, sub-planar body extensions allowing flapping flight. Given these premises, the evolution of structural elements aiming at reinforcing these extensions could then be expected. However, this anticipation barely applies to the variety of vertebrate flight organs: no oblique/transversal new bones ever evolved to address biomechanical constraints experienced by wings of pterodactyls, bats or birds. The very specific design of insect wings actually composes a limiting condition to the observed convergent regularity. The question is then whether a ‘network-and-membrane’ wing design is likely to evolve iteratively. Organismic evolution on Earth suggests that it is not: within terrestrial arthropods sharing with insect a tracheal system and an exoskeleton, which can be regarded as prerequisites for the ‘network-and-membrane’ design, none evolved flapping flight but insects. The case is therefore best characterized as of local scope, and to be related with strong selective pressure inherent to the niche of high-performance aerial predator. The case then best compares with the repeated acquisition of the dolphinoid shape [47,49].

### 4.2. Wing shape

As pointed out by Dijkstra [9] (and see [50,51]), vein reduction in petiolate wing bases impacted MP and all veins posterior to it convergently in different lineages of damselflies previously regarded as composing a single ‘Protoneuridae’ group. The view that petiolation constrain wing venation in a consistent, repeatable pattern is corroborated at a broader scale by the fossil record of Odonata [12]. Fundamentally, posterior-most veins reduced successively, from the anterior anal vein (AA), then CuP and, to some extent, CuA, or even MP. In the course of odonate evolution, petiolation very generally co-occurred with a high aspect ratio (i.e. long and narrow wings), resulting in a butter knife shape. The Triassic Triadophlebiomorpha, with their petiolate wing base and, for the largest representatives, a wingspan exceeding 30 cm [21], compose one of the rare counter-examples, as they retained a very large CuA–AA area (figure 4).

The butter knife shape habitus can be easily appreciated from the extent of the postero-basal area delimited by CuA and AA (in figure 4, CuA–AA area in magenta; area between AA and posterior wing margin in light blue). The newly discovered species, *Haidilaozhen cuiae*, provides a new case of butter knife shape, as early as in the Pennsylvanian. Other well-known instances are the Late Palaeozoic and mid-Mesozoic Archizygoptera [11,52,53] and Zygoptera (but for Polythoridae and Calopterygidae, in which the petiolate was lost), which crown-group appeared in the early Mesozoic [13]. *Haidilaozhen cuiae* is therefore among the earliest cases. Similar, sub-contemporaneous forms include *Bechlya ericrobinsoni* Jarzembowski & Nel, 2002, known from the basal half of a single wing; *Bechala sommeri* Ilger & Brauckmann, 2012, with two incomplete wings; and the enigmatic *Sowiakala perprocera* Zessin, Brauckman & Leipner, 2021, with the basal two-thirds of a single wing. The affinities of these poorly known and rare taxa remain unclear. As far as they can be compared, *Haidilaozhen cuiae* demonstrates a more definite butter knife habitus, with a more reduced CuA, CuP and AA.

The benefits of petiolate wing base on flight performance, such as flight speed, agility and manoeuvrability, remain poorly understood [54]. Indeed, the complex interactions between wing morphology and the various flight modes odonates adopt pending the context, such as foraging, escape and male–male confrontation modes, are yet to be disentangled. Moreover, flight kinematics likely play an important role, for fore- and hindwing likely perform differently [55,56]. Nevertheless, it is generally admitted that petiolate wings allow slow flight speed owing to their higher torsional compliance, making it possible to generate lift during both down- and upstroke [54,57]. The sub-parallel arrangement of longitudinal vein in conjunction with the type and distribution of vein joints is also believed to enhance camber generation [46]. In extant odonates petiolate and narrow wings commonly occur in small species, for which hovering should be comparatively energy-effective compared to larger species [54]. In summary, the iterated acquisition of butter knife-shaped wings can be reasonably related to functional aspects and flight performance.

This iterated regularity falls within the range of the precise cases [47]. And even though distinct lineages, within Odonata but also across insects [12], acquired a butter knife wing shape, these instances likely involved sub-identical developmental mechanisms (i.e. the observed instances have a low independence). However, unlike convergence relating to wing structuring elements, the butter knife shape has a more universal scope. Provided a ‘network-and-membrane’ wing design, it is an energy-efficient configuration to reach slow flight speeds, and may increase survival (i.e. predator avoidance [55]). Concurrently, aerial predators are a predictable outcome of the appearance of flight. It is noticeable that, just as for *Haidilaozhen cuiae*, the extinct Pennsylvanian sap-sucking Megasecoptera had, for their vast majority, butter-knife-shaped wings [58]. Incidentally, all these insects were likely subjected to predation by the broad-winged griffenflies, prevalent at the time. Nonetheless, the appearance of butter-knife-shaped wings seems deeply constrained by the underlying ‘network-and-membrane’ wing design. Indeed, this planform never arose among flying vertebrates.

## Data Availability

All data supporting this article have been included in the paper. The data are provided in electronic supplementary material [59] and from the Dryad Digital Repository: https://doi.org/10.5061/dryad.2547d7wwx [28].
